# A Timely Administration of Antenatal Steroids Is Highly Protective Against Intraventricular Hemorrhage: An Observational Multicenter Cohort Study of Very Low Birth Weight Infants

**DOI:** 10.3389/fped.2022.721355

**Published:** 2022-03-16

**Authors:** Ingmar Fortmann, Luisa Mertens, Hannah Boeckel, Berthold Grüttner, Alexander Humberg, Mariana Astiz, Claudia Roll, Isabell Rickleffs, Achim Rody, Christoph Härtel, Egbert Herting, Wolfgang Göpel, Verena Bossung

**Affiliations:** ^1^Department of Pediatrics, University of Lüebeck, Lübeck, Germany; ^2^German Center for Infection Research (DZIF), Partner Site Hamburg-Lübeck-Borstel-Riems, Lübeck, Germany; ^3^Department of Obstetrics and Gynecology, University of Lübeck, Lübeck, Germany; ^4^Department of Obstetrics and Gynecology, University of Cologne, Cologne, Germany; ^5^Center of Brain, Behavior and Metabolism, Institute of Neurobiology, University of Lübeck, Lübeck, Germany; ^6^Department of Pediatrics, Vestisch Children's Hospital Datteln, University Witten/Herdecke, Datteln, Germany; ^7^Department of Pediatrics, University Hospital of Würzburg, Würzburg, Germany

**Keywords:** antenatal steroids, intraventricular hemorrhage, VLBWI, preterm birth, neonatal outcome

## Abstract

**Aim:**

The aim of the study is to evaluate the influence of the timing of antenatal steroids (ANSs) on neonatal outcome of very low birth weight infants (VLBWI) born before 30 weeks of gestation in the German Neonatal Network.

**Methods:**

The German Neonatal Network is a large population-based cohort study enrolling VLBWIs since 2009. We included 672 neonates, who were born between January 1, 2009 and December 31, 2019 in our analysis in 10 selected centers. Infants were divided into four subgroups based on the interval between the first steroid administration and preterm birth: (I) two doses of betamethasone, ANS-birth interval: >24 h to 7 days, *n* = 187, (II) only one dose of betamethasone, ANS-birth interval 0–24 h, *n* = 70, (III) two doses of betamethasone, ANS-birth interval >7 days, *n* = 177, and (IV) no antenatal steroids, *n* = 238. Descriptive statistics and logistic regression analyses were performed for the main neonatal outcome parameters. Group IV (no ANS) was used as a reference.

**Results:**

An ANS-birth interval of 24 h to 7 days after the first dose was associated with a reduced risk for intraventricular hemorrhage (OR 0.17; 95% CI 0.09–0.31, *p* < 0.001) and mechanical ventilation (OR 0.37; 95% CI 0.23–0.61, *p* < 0.001), whereas the group of infants that only received a single dose of steroids reflected a subgroup at high risk for adverse neonatal outcomes; an ANS-birth interval of >7 days was still associated with a lower risk for intraventricular hemorrhage (OR 0.43; 95% CI 0.25–0.72, *p* = 0.002) and the need for mechanical ventilation (OR 0.43; 95% CI 0.27–0.71, *p* = 0.001).

**Conclusion:**

Our observational data indicate that an ANS-birth interval of 24 h to 7 days is strongly associated with a reduced risk of intraventricular hemorrhage in VLBWIs. Further research is needed to improve the prediction of preterm birth in order to achieve a timely administration of antenatal steroids that may improve neonatal outcomes such as intraventricular hemorrhage.

## Introduction

Preterm birth affects 10% of all children born worldwide with rates significantly varying between countries and continents (1). Very low birth weight infants (VLBWIs, birthweight <1,500 g) and extreme premature neonates are the most critical patients. Several determinants for neonatal outcome have been identified, including gestational age, birth weight, gender, single/multiple gestation, place of birth, mode of delivery, and antenatal exposure to corticosteroids (2–4). Several decades ago, the administration of antenatal steroids (ANS) has been proven to reduce neonatal morbidity and mortality (5). Numerous studies have confirmed these findings and showed that neonatal complications like respiratory distress syndrome (RDS), necrotizing enterocolitis (NEC), intraventricular hemorrhage (IVH), and death are reduced when steroids are given to mothers before birth (6–9). The effect of ANS is strongest in VLBWIs and infants at early gestational ages (10, 11). According to international guidelines, pregnant women before 34 weeks of gestation with imminent preterm birth within the next 7 days should receive ANS (12–15). There is a therapeutic window for the optimal effect of ANS, which is reported to be the first week after a complete cycle (8, 16), but even the newest Cochrane Review on this topic states that “further information is required for the optimal dose-to-delivery interval” (9). Recent data show that there might be early effects of ANS even hours after the initial dose (16). After a maximum of 14 days, the therapeutic effect of ANS is significantly reduced (17, 18). A reliable prediction of preterm birth is required for a timely administration of ANS. As preterm birth has many causes ranging from spontaneous preterm labor and premature rupture of membranes to maternal and fetal pathologies, such as preeclampsia or fetal growth restriction (19), its reliable prediction is rather challenging for clinicians. Several studies have demonstrated that only 20 to 50% of mothers receive ANS within 1 week before preterm birth (20–24). However, clinical trials evaluating the effects of different treatments or determinants on neonatal outcome do not commonly report the ANS-birth interval. Thus, it often stays unclear whether a therapeutic window of ANS application influences the outcome. The objective of this study was to evaluate the impact of the ANS-birth interval on infant survival and morbidity in a multicenter cohort of VLBWIs.

## Materials and Methods

### Cohort Data Collection

The German Neonatal Network (GNN) is a population-based observational multicenter cohort study of VLBWIs with a gestational age between 22/0 and 36/6 weeks enrolled in 65 German neonatal intensive care units since 2009. After obtaining written informed consent from parents or legal guardians, predefined data on general neonatal characteristics, antenatal and postnatal treatment, and outcome are recorded for each patient on clinical record files at the participating centers. After discharge, datasheets are sent to the study center (University of Luebeck). A physician or study nurse from the central GNN study office (University of Luebeck) with expertise in neonatology monitors the data quality of completed record files through annual on-site visits. As part of the standardized annually monitored documentation, the GNN record files include information on whether ANS was given to the mothers prenatally (no/complete/incomplete). However, an exact documentation of the injection timepoint is not recorded. Therefore, we performed an additional on-site monitoring of the exact timing of maternal ANS application in 10 randomly chosen GNN centers to calculate the ANS-birth interval. During this audit, we monitored maternal medical records of GNN cases with delivery and discharge between January 1, 2009 and December 31, 2019. The additional dataset included dates and times of ANS administration as well as the substance and dosage that were used (betamethasone or dexamethasone) and the number of applications. All recorded data were entered into a database, double checked, and imported into the GNN database at the study center in Luebeck.

### Inclusion and Subclass Definitions

For the current analysis, we used data collected from VLBWIs born and discharged in GNN centers between January 1, 2009 and December 31, 2019, with a gestational age between 23/0 and 29/6 weeks who received active perinatal care. By performing an extra on-site monitoring to record exact dates and times of maternal ANS administration, we included mother–infant pairs from 10 GNN centers. The centers were chosen by a quasi-randomization based on the accessibility and the possibility of our study team to perform an extra on-site monitoring of maternal charts. All mother–infant pairs, of whom dates and times of ANS administration could be extracted from the patient charts and who fulfilled our inclusion criteria, were included into our analysis. Following international standards, two doses of betamethasone were administered intramuscularly with a dose of 12 mg at an interval of 24 h. To achieve a homogenous cohort and to reduce possible confounding factors, mother–infant pairs who received more than one cycle of ANS or were treated with dexamethasone were excluded. Furthermore, infants with lethal malformations were excluded. Since information on whether ANS were administered is part of the routine GNN documentation (independent of the additional monitoring), all infants from the 10 GNN centers without ANS who met the inclusion criteria were included as a reference group in our analysis.

To evaluate the effects of the timing of ANS on neonatal outcome, four subgroups were defined based on the ANS-birth interval.

Group I: Two doses of betamethasone were administered at an interval of 24 h. The ANS-birth interval between the first application of betamethasone and preterm birth was ≤ 7 days and ≥24 h.

Group II: Only one dose of betamethasone was administered. Accordingly, the ANS-birth interval was <24 h.

Group III: Two doses of betamethasone were administered at an interval of 24 h. The ANS-birth interval between the first application of betamethasone and preterm birth was >7 days.

Group IV was the no ANS group (reference): No ANS was administered.

### Definitions

Gestational age was calculated from the best obstetric estimate based on early prenatal ultrasound and obstetric examination. Small-for-gestational age (SGA) was defined as a birth weight below the 10th percentile for gestational age according to gender-specific standards for birthweight by gestational age in Germany (25). Inotropes refer to the initial resuscitation. Clinical sepsis was defined as a condition with at least two signs of systemic inflammatory response (temperature >38°C or <36.5°C, tachycardia >200/min, new onset or increased frequency of bradycardias or apneas, hyperglycemia >140 mg/dl, base excess <-10 mval/l, changed skin color, increased oxygen requirements), one laboratory sign (e.g., C-reactive protein > 20 mg/L, immature/total neutrophil ratio > 0.2, white blood cell count <5/nl), and the neonatologist's decision to treat with anti-infective drugs for at least 5 days but with no proof of causative agent in blood culture (26). Blood culture-confirmed sepsis was defined as clinical sepsis with proof of causative agents in the blood culture. If coagulase-negative *staphylococci* (CoNS) were isolated as a single pathogen in one peripheral blood culture, two clinical signs and one laboratory sign were required for classification of CoNS sepsis (26). Bronchopulmonary dysplasia (BPD) was diagnosed when needing supplemental oxygen or ventilatory support at 36 weeks of postmenstrual age (27). NEC was defined as necrotizing intestinal inflammation requiring surgery (28). Death was defined as all-cause mortality occurring during primary stay in hospital. Intraventricular hemorrhage (IVH) grades I–IV were diagnosed according to the ultrasound criteria of Papile et al. (29). Periventricular leukomalacia (PVL) was defined as white matter brain injury characterized by cystic degeneration of white matter near the lateral ventricles as diagnosed by ultrasound imaging (30). Anencephaly, hypoplastic left heart syndrome, bilateral renal agenesis, Potter sequence, trisomy 13, and trisomy 18 were defined as lethal malformations.

### Statistical Analyses

Data analyses were performed using the SPSS 26.0 data analysis package (Munich, Germany). Hypotheses in the univariate analysis were evaluated with Pearson's Chi-square test for categorical variables and Mann–Whitney U-test for continuous variables. After univariate analyses, we performed linear and logistic regression models and included known confounders of neonatal outcomes as independent variables: gestational age per week, gender, multiple birth, mode of delivery, and SGA status. Effect size and 95% confidence intervals (CIs) were calculated. A *p*-value of <0.05 was considered statistically significant. To address the problem of multiple comparisons, we performed Bonferroni corrections for multivariate analyses in order to avoid statistical Type I errors. Additional information derived from Bonferroni correction is indicated in the Table 1 accordingly.

**Table 1:** Associations between ANS timing and neonatal outcomes (reference: no ANS group).

	**I** **24 h−7 days**	**II** **0–24 h (single dose)**	**III** **>7 days**
**Outcome**	**Odds ratio (95% CI)**		
IVH, all grades	**OR 0.17** **(0.09–0.31)** ***p*** **<** 0.001^*^	OR 0.76 (0.4–1.4) *p* = 0.4	**OR 0.43** **(0.25–0.72)** ***p*** **=** 0.002^*^
IVH II	**OR 0.18** **(0.06–0.5)** ***p*** **=** 0.003^*^	OR 0.98 (0.4–2.4) *p* = 0.96	OR 0.56 (0.2–1.4) *p* = 0.2
IVH III	OR 0.59 (0.17–1.9) *p* = 0.39	OR 0.86 (0.2–3.4) *p* = 0.83	OR 0.6 (0.17–1.9) *p* = 0.43
IVH IV	**OR 0.15** **(0.04–0.53)** ***p*** **=** 0.003^*^	OR 1.03 (0.3–3.1) *p* = 0.9	OR 0.37 (0.13–1.07) *p* = 0.07
Mechanical ventilation (in the first 72 h)	**OR 0.40** **(0.25–0.64)** ***p*** **<** 0.001^*^	OR 1.14 (0.6–2.2) *p* = 0.7	**OR 0.46** **(0.29–0.73)** ***p*** **=** 0.001^*^
Mechanical ventilation (primary stay)	**OR 0.37** **(0.23–0.61)** ***p*** **<** 0.001^*^	OR 1.29 (0.64–2.6) *p* = 0.5	**OR 0.43** **(0.27–0.71)** ***p*** **=** 0.001^*^
Death (primary stay)	OR 0.49 (0.2–1.2) *p* = 0.13	OR 0.77 (0.24–2.5) *p* = 0.67	OR 1.6 (0.6–4.5) *p* = 0.33
BPD	OR 0.78 (0.46–1.4) *p* = 0.4	OR 1.01 (0.5–2.07) *p* = 0.96	OR 1.2 (0.7–2.2) *p* = 0.44
PVL	OR 0.15 (0.02–1.25) *p* = 0.08	OR 0.64 (0.13–3.2) *p* = 0.58	OR 0.76 (0.2–2.85) *p* = 0.7

*Logistic regression models were used and adjusted for gestational age, gender, mode of delivery, SGA, multiples. Outcomes are reported as odds ratios (OR) and 95% confidence intervals. Reference category for the analysis was “no ANS”*.

*ANS, antenatal steroids; IVH, intraventricular hemorrhage; BPD, bronchopulmonary dysplasia; PVL, periventricular leukomalacia*.

* *Bonferroni correction did not change the significance of the p-value. Significant Values are in bold*.

For primary and subgroup analyses, we used a uniform dataset with available data for all metric parameters. Missing data were not included. For our statistical analyses, we defined the largest group (no ANS) as reference in the regression model.

### Ethical Approval

All study parts were approved by the University of Luebeck Ethical Committee and the committees of the participating centers (vote no. 08–022). Informed consent was obtained from all subjects. All methods were carried out in accordance with relevant guidelines and regulations specifically The Declaration of Helsinki, the current revision of ICH Topic E6, the Guidelines for Good Clinical Practice, and the Guidelines of the Council for International Organization of Medical Sciences, the World Health Organization (“Proposed International Guidelines for Biomedical Research Involving Human Subjects”).

## Results

### Cohort Characteristics at Baseline

**Figure 1:**
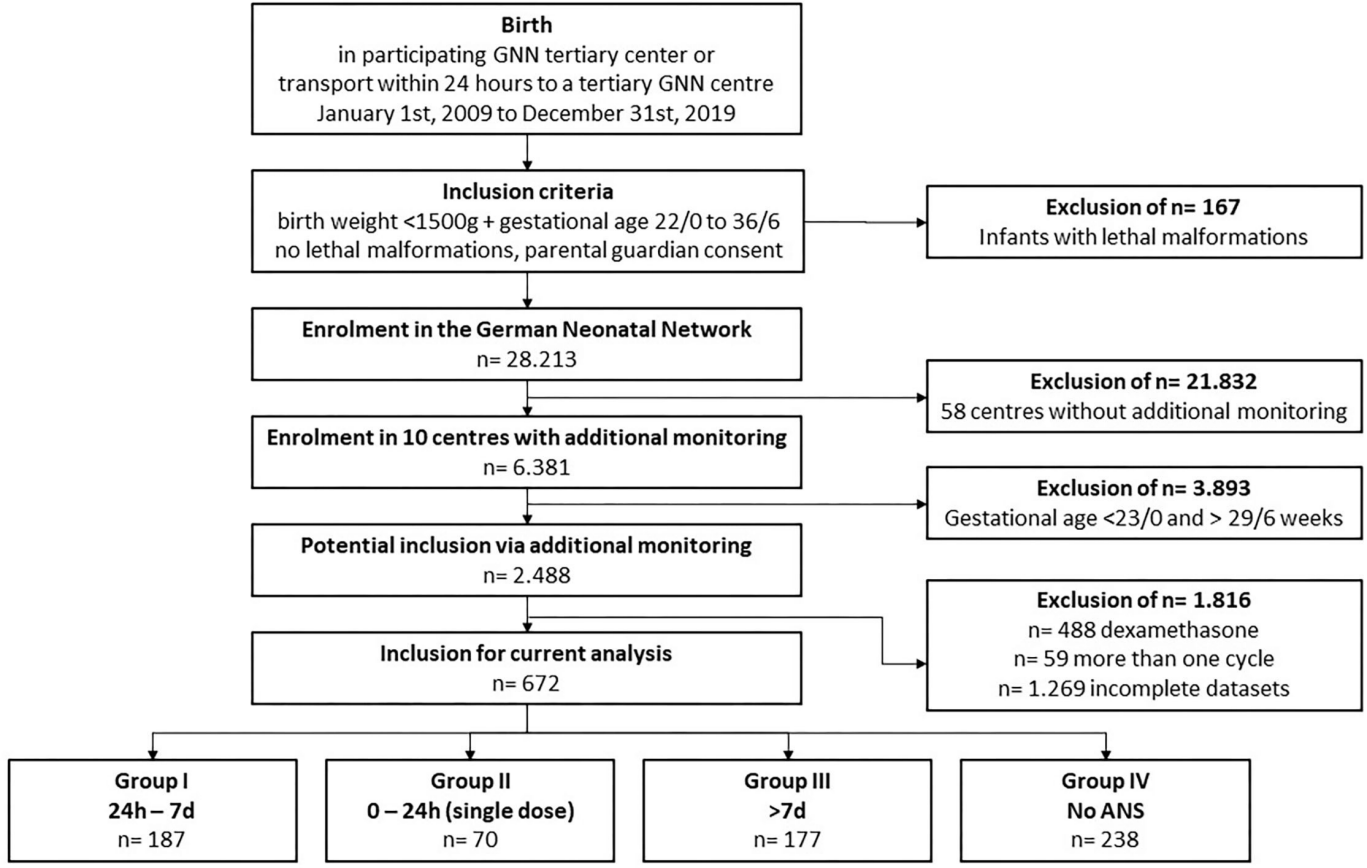
Study flowchart.

We included 672 VLBWIs in our analysis, who were born at a GA from 23/0 to 29/6 weeks between January 1, 2009 and December 31, 2019, in 10 selected GNN centers (Figure 1). The study cohort had a median gestational age at birth of 27.0 weeks [interquartile range (IQR) 25.3–28.4 weeks] and a median birth weight of 900 g (IQR 690–1,140 g; see Table 2). In our cohort, 57.2% of the infants were male, 29.4% were multiples, 10.9% were born SGA, and 10.1% were born vaginally. A total of 238 VLBWIs were not exposed to ANS (no ANS group IV), *n* = 187 received two doses of ANS 24 h to 7 days before preterm birth (group I), *n* = 70 infants had only one dose of ANS (group II), and *n* = 177 VLBWIs were treated with an ANS-birth interval of >7 days (group III; Figure 1). In group I, the ANS-birth interval was 3.8 days (IQR 2.5–5.3 days); in group III, it was 20.1 days (IQR 12.9–25.8 days). In group II, the ANS-birth interval was calculated in hours with a median of 8.0 h (IQR 4.7–13.0 ). Supplementary Figure 1 depicts all ANS-birth intervals in relation to gestational age. As the number of included infants in the subgroup without ANS was independent of the additional monitoring (data already collected within the GNN study), the proportion between ANS-exposed and -unexposed infants does not represent epidemiological proportions. Therefore, the subgroup without ANS from the 10 GNN centers is larger than usual within our study (187/672 infants, 27.8%).

**Table 2:** Cohort characteristics by ANS timing groups.

	**I** **24 h−7 days**	**II 0–24 h** **(single dose)**	**III** **>7 days**	**IV** **No ANS**	**Total**
	***n* = 187**	***n* = 70**	***n* = 177**	***n* = 238**	***n* = 672**
ANS-birth interval [median, (IQR)]	3.8 days [2.5–5.3]	8.0 h [4.7–13.0]	20.1 days [12.9–25.8]	**-**	**-**
**Characteristics**	**Median (IQR)**				
Gestational age (weeks)	26.7 [24.9–28.3] *p* = 0.55	26.7 [24.9–28.6] *p* = 0.7	**27.7** **[26.4–28.7]** ***p*** **<** **0.001**	26.6 [24.9–28.1]	27.0 [25.3–28.4]
Birth weight (grams)	**824** **[640–1,066]** ***p*** **=** **0.03**	810 [630–1,045] *p* = 0.2	**975** **[810–1,190]** ***p*** **=** **0.002**	900 [680–1,170]	900 [690–1,140]
	**% (95% CI)**				
Gender (male)	53.5 (46.3–60.5) *p* = 0.4	54.2 (42.6–65.6) *p* = 0.8	60.5 (53.1–67.4) *p* = 0.6	58.0 (51.6–64.1)	57.2 (53.8–60.9)
Multiples	23.0 (17.4–29.4) *p* = 0.9	**35.7** **(25.2–47.3)** ***p*** **=** **0.01**	**45.8** **(38.5–53.1)** ***p*** **<** **0.001**	21.4 (16.6–27.0)	29.4 (26.1–33.0)
SGA	**17.1** **(12.2–23.0)** ***p*** **=** **0.002**	**14.3** **(7.6–23.9)** ***p*** **=** **0.03**	7.9 (4.6–12.6) *p* = 0.8	7.1 (4.4–10.9)	10.9 (8.8–13.5)
Vaginal birth	**6.4** **(3.6–10.6)** ***p*** **<** **0.001**	**7.1** **(2.8–14.9)** ***p*** **=** **0.009**	**5.1** **(2.5–9.1)** ***p*** **<** **0.001**	16.0 (11.8–21.1)	10.1 (8.0–12.5)
Elective cesarean section	**86.6** **(81.2–90.9)** ***p*** **<** **0.001**	**72.9** **(61.7–82.2)** ***p*** **=** **0.009**	**83.1** **(77.0–88.0)** ***p*** **<** **0.001**	49.4 (43.0–55.7)	70.4 (66.8–73.7)
Emergency cesarean section	**7.0** **(4.0–11.3)** ***p*** **<** **0.001**	**20.0** **(12.0–30.5)** ***p*** **=** **0.009**	**11.9** **(7.7–17.2)** ***p*** **<** **0.001**	34.6 (28.8–40.8)	19.6 (16.7–22.7)

*p-Values were derived from Pearson's chi-square test for categorical variables and Mann–Whitney U-test for continuous variables. Group IV was used as a reference group for p-values*.

*ANS, antenatal steroids; IQR, interquartile range; CI, confidence interval; SGA, small for gestational age. Significant Values are in bold*.

The neonatal characteristics at baseline were different between the four groups (Table 2). VLBWIs from group III had a higher gestational age (median 27.7 weeks; IQR 26.4–28.7 weeks) and birthweight (median 975 g; IQR 810–1,190 g) than infants in all other subgroups (Table 2). Multiples were most frequent in groups II and III. Infants in groups I and II had a higher SGA rate than infants in groups III and IV. VLBWIs who were not exposed to ANS (no ANS group IV) were more frequently born vaginally or by emergency cesarean section compared with all the other groups. In the one-dose group II, 14/70 infants (20%) were born by emergency cesarean section.

### Neonatal Treatment Characteristics Stratified to Antenatal Steroids Timing Groups

In univariate analyses, VLBWIs with two doses of ANS (groups I and III) were characterized by improved respiratory characteristics and superior postnatal adaptation when compared with infants with one dose of betamethasone or no ANS (Table 3). During resuscitation, timing group I and timing group III infants were less frequently intubated when compared with infants with incomplete or no ANS [group I: 20.0% (95% CI 13.5–28.0) and group III: 26.9% (95% CI 18.7–36.5) vs. single-dose group II: 48.1% (95% CI 34.9–61.5) and no ANS group IV: 43.0% (95% CI 35.3–51.0)]. Infants with two doses of ANS (groups I and III) also had a higher rate of treatment with surfactant via LISA (less invasive surfactant application) or no surfactant compared with the other groups. Cardiopulmonary resuscitation was less frequent in group I infants compared with all other subgroups. The use of inotropes during postnatal adaptation was less common in group I than in groups II and III. Group I and group III infants had fewer rates of 5-min APGAR scores <7 and a reduced need of mechanical ventilation in the first 72 postnatal hours and during primary hospital stay. Additional oxygen supplementation in the first 12 h after birth was highest in infants with only one dose of corticosteroids when compared with all other subgroups. Infants with incomplete or no ANS were more often treated with high-frequency oscillatory ventilation (HFO) compared with infants from groups I and III.

**Table 3:** Neonatal treatment characteristics stratified by ANS timing group.

	**I** **24 h−7 days**	**II** **0–24 h** **(single dose)**	**III** **>7 days**	**IV** **No ANS**	**Total**
	***n* = 187**	***n* = 70**	***n* = 177**	***n* = 238**	***n* = 672**
**Characteristics**	**Median [IQR]**				
Umbilical cord blood pH (arterial)	**7.36 ** **[7.30–7.38]** ***p*** **<** **0.001**	7.32 [7.27–7.37] *p* = 0.34	**7.36** **[7.31–7.39]** ***p*** **<** **0.001**	7.33 [7.28–7.39]	7.31 [7.25–7.37]
Maximum O_2_ need (first 12 h)	40.0 [30.0–50.0] *p* = 0.2	**50.0** **[35.0–80.0]** ***p*** **=** **0.03**	40.0 [30–60] *p* = 0.3	40.0 [30.0–65.0]	40.0 [30.0–60.0]
	**% (95% CI)**				
Intubation (during resuscitation)	**20.0** **(13.5–28.0)** ***p*** **<** **0.001**	48.1 (34.9–61.5) *p* = 0.5	**26.9** **(18.7–36.5)** ***p*** **=** **0.01**	43.0 (35.3–51.0)	33.7 (29.2–38.3)
Mechanical ventilation (first 72 h of life)	**45.5** **(38.4–52.6)** ***p*** **<** **0.001**	64.3 (52.7–74.8) *p* = 0.9	**37.3** **(30.4–44.6)** ***p*** **<** **0.001**	63.9 (57.5–69.9)	51.6 (47.8–55.4)
Surfactant via LISA (during resuscitation)	**54.5** **(47.4–61.6)** ***p*** **<** **0.001**	41.4 (30.0–53.1) *p* = 0.7	**54.2** **(46.9–61.5)** ***p*** **<** **0.001**	35.7 (29.7–42.1)	46.6 (42.8–50.4)
No surfactant (during resuscitation)	**15.5** **(10.9–21.2)** ***p*** **<** **0.001**	11.4 (5.6–20.4) *p* = 0.15	**18.6** **(13.4–24.9)** ***p*** **<** **0.001**	12.8 (8.9–17.6)	14.9 (12.3–17.7)
CPR (resuscitation)	3.4 (1.2–8.0) *p* = 0.06	15.4 (7.6–26.9) *p* = 0.22	10.8 (5.7–18.2) *p* = 0.7	9.3 (5.4–14.7)	8.8 (6.3–11.8)
Inotropes (resuscitation)	15.5 (10.9–21.2) *p* = 0.9	22.7 (13.9–33.3) *p* = 0.21	20.8 (15.3–27.3) *p* = 0.7	14.9 (10.3–20.6)	15.0 (12.1–18.3)
APGAR 5 <7	**19.4** **(14.2–25.5)** ***p*** **<** **0.001**	25.7 (16.6–36.8) *p* = 0.13	**16.4** **(11.5–22.4)** ***p*** **<** **0.001**	35.3 (29.2–41.8)	24.7 (21.5–28.1)
HFO	**15.9** **(9.4–24.6)** ***p*** **=** **0.003**	20.9 (9.9–34.2) *p* = 0.09	**6.5** **(3.0–12.4)** ***p*** **<** **0.001**	34.6 (26.1–43.9)	19.3 (15.4–23.7)

*p-Values were derived from Pearson's chi-square test for categorical variables and Mann–Whitney U-test for continuous variables. Group IV was used as a reference group for p-values*.

*ANS, antenatal steroids; CI, confidence interval; LISA, less invasive surfactant application; CPR, cardiopulmonary resuscitation; HFO, high-frequency oscillatory ventilation*.

*Significant Values are in bold*.

### Neonatal Outcomes in Univariate Analysis Stratified to Antenatal Steroids Timing Groups

**Figure 2:**
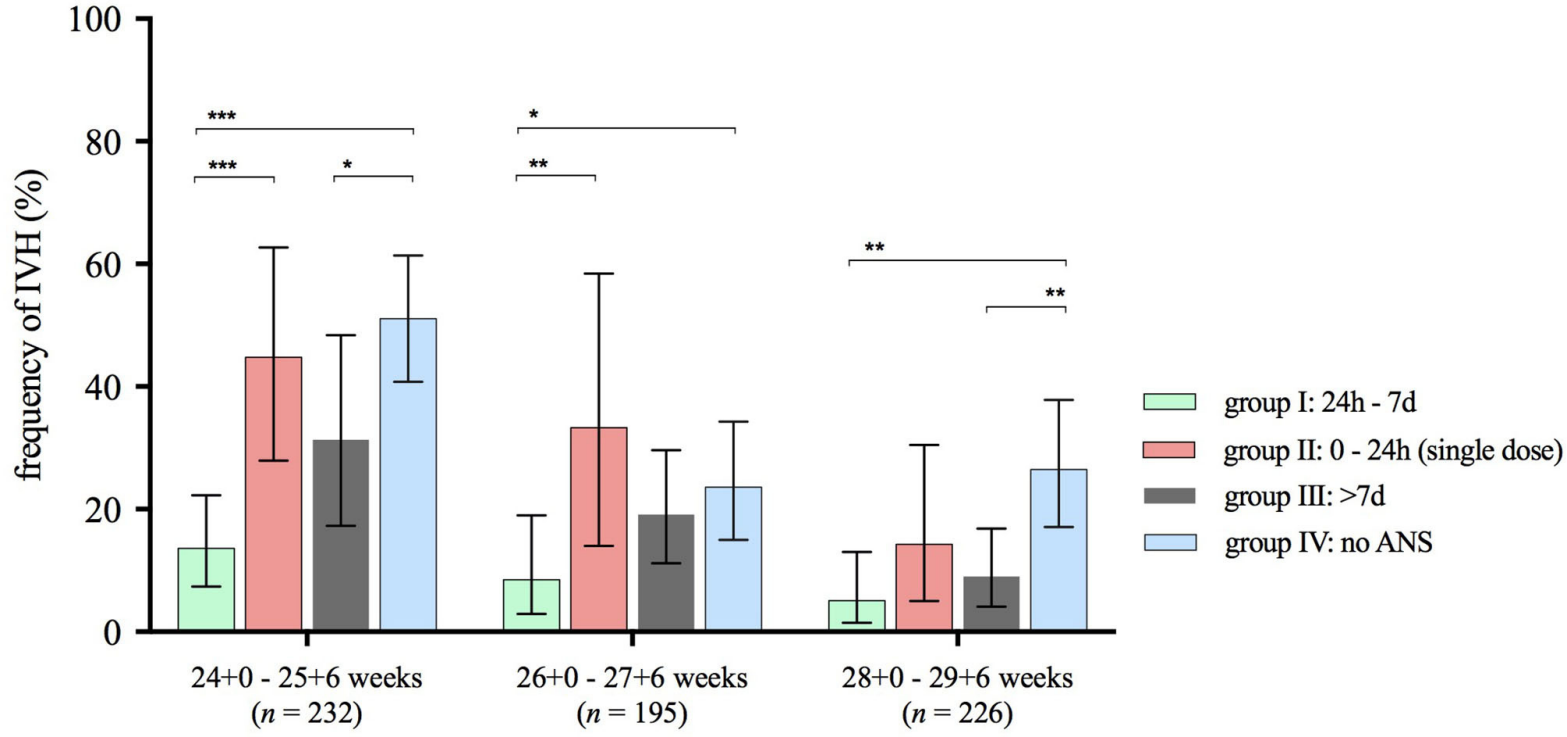
Frequency of IVH stratified to ANS exposure groups and gestational age subgroups. ANS, antenatal steroids; IVH, intraventricular hemorrhage. Horizontal lines connect subgroups with significant differences; ^*^p < 0.05, ^**^p < 0.01, ^***^p < 0.001; error bars show 95% confidence intervals. Number n represents the size of analyzed subcohort in respective gestational age range.

**Figure 3:**
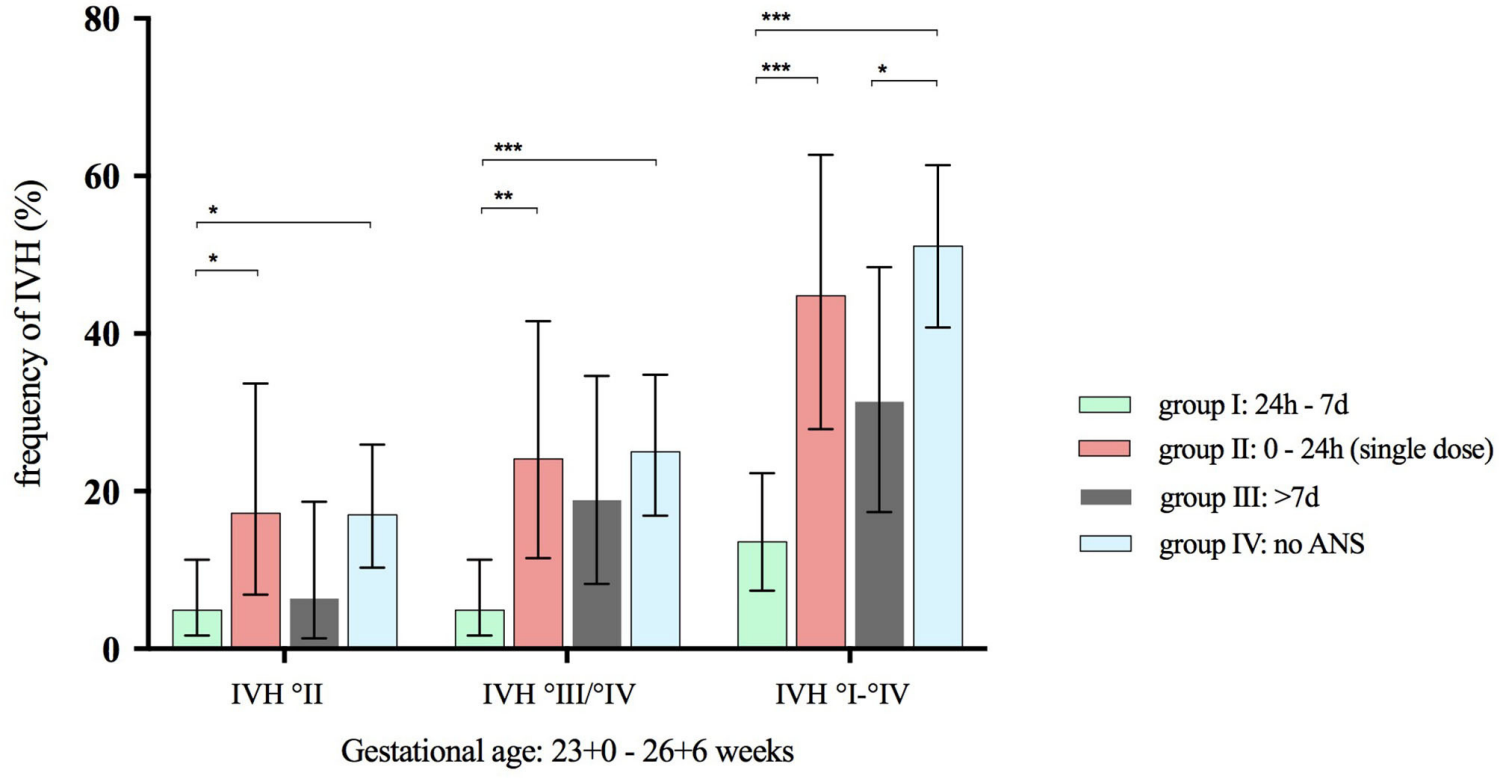
Frequency of IVH grades stratified to ANS exposure groups. ANS, antenatal steroids; IVH, intraventricular hemorrhage. Horizontal lines connect subgroups with significant differences; ^*^p < 0.05, ^**^p < 0.01, ^***^p < 0.001; error bars show 95% confidence interval; *n* = 309 infants with a gestational age from 23 + 0 to 26 + 6.

Univariate analyses of major neonatal morbidities and mortality demonstrated significant differences between the four timing groups, mainly regarding the rate of IVH. In our cohort, the overall rate of IVH was 22.0% (95% CI 19.0–25.3). IVH was almost four times as frequent in infants without ANS [34.5% (95% CI 28.5–40.9) compared with 9.1% in group I (95% CI 5.6–13.8), *p* < 0.001], more than three times as frequent in infants with a single dose of betamethasone [30.0% (95% CI 20.2–41.4), *p* = 0.04] and almost twice as frequent in group III [16.4% (95% CI 11.5–22.4), *p* < 0.001] when compared with infants in group I (Table 4). Figures 2, 3 illustrate the reduced rates for IVH by two doses of ANS in univariate analyses. They demonstrate that IVH was least frequent in infants of group I with an ANS-birth interval of 24 h to 7 days throughout all gestational age groups (Figure 2). This was confirmed in VLBWIs at highest risk for IVH due to extreme prematurity, such as infants with a gestational age between 23/0 and 26/6 weeks (Figure 3).

**Table 4:** Neonatal outcomes stratified to ANS stratified by ANS timing group.

	**I** **24 h−7 days**	**II** **0–24 h (single dose)**	**III** **>7 days**	**IV** **No ANS**	**Total**
	***n* = 187**	***n* = 70**	***n* = 177**	***n* = 238**	***n* = 672**
**Outcome**	**% (95% CI)**				
Culture-positive sepsis	14.4 (10.0–20.0) *p* = 0.5	15.7 (8.6–25.5) *p* = 0.8	16.9 (12.0–23.0) *p* = 0.9	16.7 (12.3–22.0)	16.1 (13.4–19.0)
IVH	**9.1** **(5.6–13.8)** ***p*** **<** **0.001**	**30.0** **(20.2–41.4)** ***p*** **=** **0.04**	**16.4** **(11.5–22.4)** ***p*** **<** **0.001**	34.5 (28.5–40.9)	22.0 (19.0–25.3)
IVH III/IV	**3.8** **(1.7–7.3)** ***p*** **<** **0.001**	12.7 (6.5–21.9) *p* = 0.78	**5.6** **(2.9–9.7)** ***p*** **=** **0.003**	14.2 (10.1–19.1)	8.8 (6.8–11.1)
PVL	**0.5** **(0.1–2.5)** ***p*** **=** **0.01**	2.9 (0.6–9.0) *p* = 0.6	2.3 (0.8–5.3) *p* = 0.2	4.4 (2.3–7.7)	2.6 (1.6–4.0)
NEC	3.2 (1.4–6.5) *p* = 0.5	**8.6** **(3.7–16.8)** ***p*** **=** **0.01**	1.1 (0.2–3.6) *p* = 0.4	2.2 (0.8–4.8)	2.9 (1.8–4.4)
BPD	20.3 (15.0–26.5) *p* = 0.7	22.9 (14.2–33.7) *p* = 0.9	16.9 (12.0–23.0) *p* = 0.2	22.0 (17.0–27.7)	20.3 (17.4–23.5)
Death (during primary stay)	4.8 (2.4–8.6) *p* = 0.2	7.1 (2.8–14.9) *p* = 0.8	4.5 (2.2–8.3) *p* = 0.2	8.0 (5.0–12.0)	6.1 (4.4–8.1)

*p-Values were derived from Pearson's chi-square test for categorical variables and Mann–Whitney U-test for continuous variables. Group IV was used as a reference group for p-values*.

*ANS, antenatal steroids; CI, confidence interval; IVH, intraventricular hemorrhage; PVL, periventricular leukomalacia; NEC, necrotizing enterocolitis; BPD, bronchopulmonary dysplasia*.

*Significant Values are in bold*.

In our cohort, 6.1% of the VLBWIs died during the primary hospital stay. Mortality was lower in group I (4.8%) and group III (4.5%) compared with group IV (8.0%) and group II (7.1%), but the difference did not reach significance (Table 4). There were no differences for BPD, FIP, and culture-proven sepsis between the groups. The single-dose group II had the highest rates of clinical sepsis (54.3%, *p* = 0.01) and NEC (8.6%, *p* = 0.01). The PVL rate was lowest in group I compared with all other subgroups [0.5% (95% CI 0.1–2.5) vs. 4.4% (95% CI 2.3–7.7), *p* < 0.001].

### Timely Application of Antenatal Steroids Reduces the Risk for Intraventricular Hemorrhage in Logistic Regression Analysis

In logistic regression models, we adjusted our results for main confounders of adverse neonatal outcomes, such as gestational age, male gender, multiple delivery, delivery mode, and SGA status (Table 1). The no ANS group IV was used as a reference. Notably, an ANS-birth interval of 24 h to 7 days after ANS was highly protective against IVH II and IV as well as all grades of IVH (OR 0.17; 95% CI 0.09–0.31, *p* < 0.001). An ANS-birth interval of more than 7 days proved to be protective against IVH with a higher OR than that for group I (OR 0.43; 95% CI 0.25–0.72, *p* = 0.002), whereas a single dose of betamethasone did not affect the IVH risk (OR 0.76; 95% CI 0.4–1.4, *p* = 0.4). When adjusted for main confounders, timely administration of ANS did not affect the infants' overall mortality (group I: OR 0.49; 95% CI 0.2–1.2, *p* = 0.13). Furthermore, logistic regression models demonstrated that ANS-birth interval of 24 h to 7 days is protective against the need for mechanical ventilation (OR 0.37; 95% CI 0.23–0.61, *p* < 0.001) but not BPD. Single doses of ANS did not have a protective effect on neonatal outcome in our cohort. An ANS-birth interval >7 days (group III) was associated with lower risks for IVH and mechanical ventilation but not BPD and PVL.

To address further potential confounders reflecting disease severity at birth and reasons of preterm birth, we performed an alternative logistic regression model in which we additionally included the parameters Apgar <7 and primary intubation that were derived from univariate analyses (Table 3; surrogate measures for severe clinical course) and amnion infection syndrome, preeclampsia, and pathological Doppler/FGR. In this model, an ANS-birth interval of 24 h to 7 days (OR 0.3; 95% CI 0.2–0.6, *p* < 0.001) and >7 days (OR 0.6; 95% CI 0.27–0.98, *p* = 0.04) remained associated with a reduced risk for all grades of IVH, whereas single-dose betamethasone did not affect the IVH risk (OR 1.2; 95% CI 0.6–2.5, *p* = 0.6). In the alternative model, mortality remained unaffected, and the risk for mechanical ventilation during primary stay was significantly reduced in group I (OR 0.52; 95% CI 0.28–0.75, *p* < 0.001) and group III (OR 0.78; 95% CI 0.36–1.61, *p* < 0.001) and not in group II (OR 1.6; 95% CI 0.83–2.59, *p* = 0.19).

In a second alternative model, we tested our primary outcome (IVH) in subgroups with different cutoffs for the ANS-birth interval in order to address clinical situations, where prolongation of the pregnancy for a couple of hours (i.e., waiting for 24 h if possible) seems feasible: (a) 0–12 h; *n* = 42, (b) 12–24 h; *n* = 28, (c) 24–48 h; *n* = 44, (d) 48 h to 7 days; *n* = 143, (e) 7–14 days; *n* = 92, (f) >14 days; *n* = 85, (e) no ANS; *n* = 238. Our alternative logistic regression model was adjusted for gestational age, gender, multiple birth, delivery mode, SGA, preeclampsia, pathological Doppler/IUGR, amnion infection syndrome, Apgar <7, and primary intubation as surrogate measure for complicated clinical course. A time interval of 48 h after first dose of betamethasone (complete cycle of ANS) to 7 days (168 h) after the first dose was associated with a lower risk for IVH (OR 0.1, 95% CI: 0.02–0.2, *p* < 0.001) as well as a time interval of 7–14 days (OR 0.16, 95% CI: 0.05–0.5, *p* = 0.001). No associations between the IVH risk and the time interval were observed in the three subgroups with an ANS-birth interval <48 h (groups a, b, and c) and the subgroup of infants in group f (>14 days). However, data must be interpreted with caution due to the low sample sizes in the subgroups.

## Discussion

In this multicenter cohort of the German Neonatal Network, we hypothesize that VLBWIs, whose mothers have received two doses of ANS with a timing of >24 h to 7 d before birth, have a reduced risk for intraventricular hemorrhage and mechanical ventilation during primary hospital stay. We suggest a protective effect of an ANS-birth interval of 24 h to 7 days on all grades of IVH (OR 0.17, CI 0.09–0.31, *p* < 0.001) and severe IVH (OR 0.15, CI 0.04–0.53, *p* = 0.003). An ANS-birth interval >7 days prior birth was also associated with a reduced risk intraventricular hemorrhage and the need for mechanical ventilation, whereas in the group of infants with a single dose of steroids, an independent association between ANS and neonatal outcomes was not measurable.

Precise timing of ANS remains a challenge for clinicians. A reliable prediction of preterm birth is a requirement to determine whether and when ANS must be applied. However, preterm birth is caused by various conditions (19), which makes its prediction difficult during medical decision making. The large group of spontaneous preterm labor is particularly difficult to characterize. In this group, the measurement of cervical length by vaginal ultrasound and different biomarker tests such as fetal fibronectin have been intensively studied in obstetrics to predict preterm birth. Although these biomarkers can identify pregnant women at risk for preterm delivery, they do not allow a precise prediction of birth within the next 7 days (31–33). Various trials were able to show that only around 40% of preterm infants born before 34 weeks of gestation had received ANS within 7 days prior to birth (21–24). Hence, the impact of the ANS-birth interval on neonatal outcome is a relevant clinical question. Although antenatal steroids have been studied for several decades, researchers have not been able to answer important questions on risks and benefits for mothers and infants of this standard prenatal intervention. Timing in relation to birth—as reported in this manuscript—is only one aspect. Recently, Astiz et al. reported on the exposure to ANS during different circadian phases and associated long-term neonatal outcome parameters (34). Their data demonstrates that ANS is associated with adverse effects on behavioral development if it is given off-phase to the mother's natural circadian corticosteroid cycle. The potential association between ANS and adverse neurodevelopment has been documented by others as well (35). This is of relevance, as many mothers who were prenatally exposed to steroids because of suspected imminent preterm birth, eventually never deliver a preterm. In a recent review, Jobe et al. highlight that—based on animal models—the current dosing of ANS used worldwide is significantly higher than needed for optimal protective effects, which in turn increases the risk of side effects (36). The authors call for clinical trials evaluating lower dosing schemes and identifying fetuses who benefit most from ANS.

Whereas our study suggests protective effects of ANS on IVH and the need for mechanical ventilation, which are influenced by its timing, other neonatal outcome parameters such as BPD, PVL, and death were unaffected. Comparable recent studies have shown inconsistent results. Norman et al. evaluated the effect of ANS on neonatal mortality and severe brain injury in a large European cohort (*n* = 4,594, 24/0–32/0 weeks) and reported reduced risks for mortality and severe neonatal brain injury, which decreased 1 week after steroid application (16). The rate of severe IVH in this study was similar to our cohort (8.9%). In contrast to our results, a single dose of ANS significantly reduced mortality (RR 0.5; 95% CI 0.4–0.6). This inconsistency might be explained by the small sample size in our study and the higher overall mortality in their cohort compared with ours (total: 11% vs. 6.1%; no ANS: 20.6% vs. 8.0%). On the other hand, infants whose mothers were only given one dose of corticosteroids before birth reflect a subgroup of preterm infants, which is frequently born in a clinical emergency setting. This is reflected by a high proportion of primary intubation, mechanical ventilation, cardiopulmonary resuscitation (CPR), and inotrope exposure during resuscitation in this subgroup. Hence, a potentially existing beneficial effect of single-dose betamethasone might be concealed by the overall higher vulnerability of these infants. Furthermore, in the presence of imminent preterm birth, a prompt initiation of ANS is essential to increase the chance of a complete course prior to delivery, the beneficial effect of which is unquestioned (37).

Liebowitz et al. studied the impact of ANS timing on neonatal outcome and specifically on intraventricular hemorrhage in infants born before 28 weeks. Similar to our cohort, single-dose ANS was associated with a higher risk of IVH and RDS and also death. Furthermore, the authors found a higher incidence of severe intraventricular hemorrhage if the ANS-birth interval exceeded 9 days (17% vs. 7%) (38), which may point toward a more detailed risk stratification within group III that remained unaddressed in our study due to the lower sample size of our cohort. Furthermore, the effect on mortality might have reached significant levels due to higher mortality in the study of Liebowitz et al. [(38)] (11.5% vs. 6.1%). Norberg et al. explored the impact of ANS timing on extremely preterm infants in Sweden (*n* = 707). They demonstrated better survival in extremely preterm infants (22 to 26 weeks), if ANS were applied 24 h to 7 days before preterm birth (39) but did not find significant survival differences in infants without major neonatal morbidity (including severe IVH). In their cohort, a single dose and an ANS-birth interval >7 days were associated with higher mortality. However, the comparability to our cohort is limited, as their cohort of very premature infants was born between 2004 and 2007 and was characterized by a relatively high mortality (30% within the first year). Another study by Frändberg et al. (22) evaluated timing of ANS and neonatal outcome in a retrospective cohort study (*n* = 498, <34/0 weeks, USA) that defined the same timing groups as we did. A higher risk for RDS was shown in the groups with an ANS-birth interval >7 days, a single dose, and no ANS, but—similar to our results—they did not observe mortality differences. IVH was only analyzed within a composite adverse outcome, which was not different between the groups. Battarbee et al. performed a large retrospective secondary analysis of two multicenter studies on mother–infant pairs with mainly spontaneous preterm birth before 34/0 weeks (40). Infants with an ANS-birth interval of 2 to <7 days had the lowest rates of RDS, and a composite outcome of severe neonatal morbidities was significantly increased in the ANS group of >14 days. However, IVH alone was not reported. Furthermore, in contrast to our study design, the authors did not include mother–infant pairs without ANS but also included neonates born >1,500 g; hence, rates of mortality and of severe IVH were comparatively low in their trial.

In conclusion, there are several factors that could explain the differences between ANS effects in the studied populations: First, the gestational age of included infants plays a major role as earlier gestational ages come with a higher risk for adverse outcomes and, therefore, a potentially greater benefit from ANS. Second, mortality rates in the different cohorts range from as low as 6.1% in our cohort to 30% in a US cohort (22). Hence, the ANS effect on mortality might be weaker in a cohort with low mortality like ours. Third, the mentioned studies were performed at various times and in different countries, which reflects remarkable variability regarding routine care and incidences of neonatal outcomes. Our data, therefore, provide a benchmark for the population-based context of German preterm infants. Consequently, we cannot conclude that there is no association between ANS and reduced mortality, but only that we were unable to show this effect in a relatively small cohort for statistical reasons. However, our analyses provide valuable data for metanalyses that may address this issue in the future.

We are aware of the strengths and limitations of our data. The major strengths are the multicenter setting and the accurate phenotypic characterization of the infants by an additional data monitoring. However, there are also some limitations: First, there were some differences at baseline clinical characteristics between the VLBWIs in the four groups concerning gestational age, birthweight, birth mode, and multiple and SGA rates. Specifically, infants in groups I and II had a higher SGA rate than infants in groups III and IV. We believe that this is attributable to the distribution of the indications for ANS in our timing groups. It has been published before that a timely administration of ANS is achieved more often for mother–infant pairs with preeclampsia, fetal growth restriction, and PPROM than for women with preterm labor (24). Hence, more growth-restricted fetuses are represented in groups I and II. To account for the differences at baseline, we adjusted our model for gestational age, gender, mode of delivery, SGA, and multiples. We also performed an alternative regression model that additionally accounts for surrogate measures for severe clinical course and causes of preterm birth, which did not change the main results of our study. Still, our cohort is heterogeneous concerning the reasons for preterm birth (spontaneous and indicated preterm birth), which could also influence neonatal outcome. Second, within the 68 multicenter GNN study, we present data from only 10 randomly selected centers with an additional monitoring. Only mother–infant pairs of whom the ANS timing was documented in the patient files could be included. This might have produced selection bias and caused an unrealistic distribution of the four timing groups. The no ANS group was, by far, the largest group of our cohort (*n* = 238/672). The proportion of infants without ANS between 23 and 30 weeks in the whole GNN is 7.5%, and in the 10 selected centers, it is 7.2%. For our statistical analyses, we defined the largest group (no ANS) as reference in the regression model. Furthermore, this subcohort represents a relatively homogenous group as it does not imply variation in ANS timing. We are aware that this group includes more emergency deliveries; thus, we adjusted our regression models for birth mode (vaginal birth, elective cesarean, or emergency cesarean section). Third, our cohort was too small to further subdivide our subgroups. The cutoffs for timing groups are set very differently by international study groups, which might explain a part of the conflicting results. To address this aspect, we calculated an alternative regression model including smaller subgroups. An ANS-birth interval of 48 h to 7 days was associated with a lower risk for IVH than an ANS-birth interval of 7–14 days. No associations between the IVH risk and the time interval were observed in the three subgroups of an ANS-birth interval of <48 h and the subgroup of infants >14 days. However, the numbers in the groups for this calculation were small. Another limitation is the fact that we only present data on short-term outcomes. Mortality could possibly be underestimated due to very early deaths if parents were not approached for consent soon enough after birth. VLBWIs without active perinatal care are not part of the GNN. The suggested influence of ANS timing on long-term neonatal outcomes (34) could not be addressed in the current analysis due to the small number of children with a 5-year follow-up (cognitive and motoric tests, lung function, visual, and hearing tests). In future studies, it is important to address long-term neonatal outcomes to explore whether the early benefits of a timely administration of ANS translate into later childhood. In addition, several factors influencing risk of adverse short-term outcomes might have been missed in our regression models.

In summary, international studies show inconclusive results concerning the impact of ANS timing on neonatal outcomes. Especially within the high-risk subcohort of preterm infants who only received one dose of ANS before birth, the relevance of incomplete steroid exposure for adverse neonatal outcome remains unclear. Data from the discussed studies suggest that highly protective effects against IVH may be achieved by optimized timing of ANS. Exposure to a complete cycle of ANS within 1 week before preterm birth of VLBWIs was strongly associated with a reduced risk for IVH, especially severe IVH. As intraventricular hemorrhage is a major contributor to long-term morbidity, achieving a timely administration of ANS should be a focus of perinatologists. Data on the impact of ANS timing on neonatal mortality are quite diverse in the cited literature, which might be due to differences in the studies' overall mortality rates. At 6.1% in total and 8.0% for the subgroup without ANS, mortality rates were relatively low in our study when compared with the overall mortality rate within the GNN (41). Furthermore, our sample size might have been not large enough to identify associations with mortality that reach statistical significance.

## Conclusion

Our study suggests that the timing of antenatal steroids has a relevant impact on neonatal morbidity of VLBWIs. An ANS-birth interval of 24 h to 7 days was associated with the lowest risk for intraventricular hemorrhage followed by an interval of >7 days. Future research is necessary to improve the prediction of preterm birth in order to achieve a timely administration of antenatal steroids for an optimal effect on neonatal outcome.

## Data Availability Statement

The raw data supporting the conclusions of this article will be made available by the authors, without undue reservation.

## Ethics Statement

The studies involving human participants were reviewed and approved by the Local Ethics Committee for Research in Human Subjects of the University of Lübeck (file number 08-022) and by the Local Ethics Committees of all participating centers has been granted. Written informed consent to participate in this study was provided by the participants' legal guardian/next of kin.

## Author Contributions

IF and VB: study concept, design, and drafting of the manuscript. All GNN sites: acquisition of data. IF and WG: statistical analyses. IF, LM, HB, BG, AH, MA, CR, IR, AR, CH, EH, WG, and VB: analysis, interpretation of data, and critical revision of the manuscript for important intellectual content. WG: obtained funding and study supervision. All authors contributed to the manuscript revision and approved the final version.

## Funding

The GNN is funded by the German Ministry for Education and Research (BMBF-grant-nos: 01ER0805 and 01ER1501). The sponsor had no role in (1) the study design; (2) the collection, analysis, and interpretation of data; (3) the writing of the report; and (4) the decision to submit the paper for publication.

## Conflict of Interest

The authors declare that the research was conducted in the absence of any commercial or financial relationships that could be construed as a potential conflict of interest.

## Publisher's Note

All claims expressed in this article are solely those of the authors and do not necessarily represent those of their affiliated organizations, or those of the publisher, the editors and the reviewers. Any product that may be evaluated in this article, or claim that may be made by its manufacturer, is not guaranteed or endorsed by the publisher.

## Supplementary Material

Supplementary Figure 1 Dot plot of ANS-birth intervals throughout different subgroups stratified by gestational age. ANS, antenatal steroids.

## Abbreviations

ANS: antenatal steroids
BPD: bronchopulmonary dysplasia
CI: confidence interval
CoNS: coagulase-negative staphylococci
CPR: cardiopulmonary resuscitation
FIP: focal intestinal perforation
GNN: German Neonatal Network
HFO: high-frequency oscillatory ventilation
IVH: intraventricular hemorrhage
IQR: interquartile range
NEC: necrotizing enterocolitis
OR: odds ratio
PVL: periventricular leukomalacia
RDS: respiratory distress syndrome
SGA: small for gestational age
VLBWI: very low birth weight infant.

## References

[1] Blencowe H Cousens S Oestergaard MZ Chou D Moller A-B Narwal R. National, regional, and worldwide estimates of preterm birth rates in the year 2010 with time trends since 1990 for selected countries: a systematic analysis and implications. Lancet. (2012) 379:2162–72. 10.1016/S0140-6736(12)60820-4 22682464

[2] Stoll BJ Hansen NI Bell EF Walsh MC Carlo WA Shankaran S. Trends in care practices, morbidity, and mortality of extremely preterm neonates, 1993-2012. JAMA. (2015) 314:1039. 10.1001/jama.2015.10244 26348753 PMC4787615

[3] Travers CP Carlo WA McDonald SA Das A Bell EF Ambalavanan N. Mortality and pulmonary outcomes of extremely preterm infants exposed to antenatal corticosteroids. Am J Obstet Gynecol. (2018) 218:130.e1–e13. 10.1016/j.ajog.2017.11.554 29138031 PMC5842434

[4] Manuck TA Rice MM Bailit JL Grobman WA Reddy UM Wapner RJ. Preterm neonatal morbidity and mortality by gestational age: a contemporary cohort. Am J Obstet Gynecol. (2016) 215:103.e1–e14. 10.1016/j.ajog.2016.01.004 26772790 PMC4921282

[5] Liggins GC Howie RN. A controlled trial of antepartum glucocorticoid treatment for prevention of the respiratory distress syndrome in premature infants. Pediatrics. (1972) 50:515–25. 10.1542/peds.50.4.515 4561295

[6] Carlo WA McDonald SA Fanaroff AA Vohr BR Stoll BJ Ehrenkranz RA. Association of antenatal corticosteroids with mortality and neurodevelopmental outcomes among infants born at 22 to 25 weeks' gestation. JAMA. (2011) 306:2348–58. 10.1001/jama.2011.1752 22147379 PMC3565238

[7] Mori R Kusuda S Fujimura M. Antenatal corticosteroids promote survival of extremely preterm infants born at 22 to 23 weeks of gestation. J Pediatr. (2011) 159:110–4.e1. 10.1016/j.jpeds.2010.12.039 21334006

[8] Roberts D Brown J Medley N Dalziel SR. Antenatal corticosteroids for accelerating fetal lung maturation for women at risk of preterm birth. Cochrane Database Syst Rev. (2017) 3:CD004454. 10.1002/14651858.CD004454.pub3 28321847 PMC6464568

[9] McGoldrick E Stewart F Parker R Dalziel SR. Antenatal corticosteroids for accelerating fetal lung maturation for women at risk of preterm birth. Cochrane Database Syst Rev. (2020) 12:CD004454. 10.1002/14651858.CD004454.pub4 33368142 PMC8094626

[10] Travers CP Clark RH Spitzer AR Das A Garite TJ Carlo WA. Exposure to any antenatal corticosteroids and outcomes in preterm infants by gestational age: Prospective cohort study. BMJ. (2017) 356:j1039. 10.1136/bmj.j1039 28351838 PMC5373674

[11] Deshmukh M Patole S. Antenatal corticosteroids for neonates born before 25 Weeks-a systematic review and meta-analysis. PLoS One. (2017) 12:e0176090. 10.1371/journal.pone.0176090 28486556 PMC5423600

[12] Berger R Abele H Bahlmann F Bedei I Doubek K Felderhoff-Müser U. Prevention and therapy of preterm birthguideline of the DGGG, OEGGG and SGGG (S2k Level, AWMF Registry Number 015/025, February 2019) - Part 1 with recommendations on the epidemiology, etiology, prediction, primary and secondary prevention of preterm birth. Z Geburtshilfe Neonatol. (2019) 223:304–16. 10.1055/a-0903-2671 31623006

[13] Di Renzo GC Cabero Roura L Facchinetti F Helmer H Hubinont C Jacobsson B. Preterm labor and birth management: recommendations from the European Association of perinatal medicine. J Matern Neonatal Med. (2017) 30:2011–30. 10.1080/14767058.2017.1323860 28482713

[14] American College of Obstetricians and Gynecologists. Committee opinion no. 713: antenatal corticosteroid therapy for fetal maturation. Obstet Gynecol. (2017) 130:e102–9. 10.1097/AOG.0000000000002237 28742678

[15] Sarri G Davies M Gholitabar M Norman JE. Preterm labour: summary of NICE guidance. BMJ. (2015) 351:h6283. 10.1136/bmj.h6283 26596828

[16] Norman M Piedvache A Børch K Huusom LD Bonamy AKE Howell EA. Association of short antenatal corticosteroid administration-to-birth intervals with survival and morbidity among very preterm infants results from the EPICE cohort. JAMA Pediatr. (2017) 171:678–86. 10.1001/jamapediatrics.2017.0602 28505223 PMC5710338

[17] Ring AM Garland JS Stafeil BR Carr MH Peckman GS Pircon RA. The effect of a prolonged time interval between antenatal corticosteroid administration and delivery on outcomes in preterm neonates: a cohort study. Am J Obstet Gynecol. (2007) 196:457.e1–e6. 10.1016/j.ajog.2006.12.018 17466700

[18] Wilms FF Vis JY Pattinaja DAPM Kuin RA Stam MC Reuvers JM. Relationship between the time interval from antenatal corticosteroid administration until preterm birth and the occurrence of respiratory morbidity. Am J Obstet Gynecol. (2011) 205:49.e1–e7. 10.1016/j.ajog.2011.03.035 21620358

[19] Villar J Papageorghiou AT Knight HE Gravett MG Iams J Waller SA. The preterm birth syndrome: a prototype phenotypic classification. Am J Obstet Gynecol. (2012) 206:119–23. 10.1016/j.ajog.2011.10.866 22177191

[20] Razaz N Skoll A Fahey J Allen VM Joseph KS. Trends in optimal, suboptimal, and questionably appropriate receipt of antenatal corticosteroid prophylaxis. Obstet Gynecol. (2015) 125:288–96. 10.1097/AOG.0000000000000629 25568996

[21] Levin HI Ananth C V Benjamin-Boamah C Siddiq Z Son M Friedman AM. Clinical indication and timing of antenatal corticosteroid administration at a single centre. BJOG An Int J Obstet Gynaecol. (2016) 123:409–14. 10.1111/1471-0528.13730 26485686

[22] Frändberg J Sandblom J Bruschettini M Maršál K Kristensen K. Antenatal corticosteroids: a retrospective cohort study on timing, indications and neonatal outcome. Acta Obstet Gynecol Scand. (2018) 97:591–7. 10.1111/aogs.13301 29360141

[23] Boesveld M Heida KY Oudijk MA Brouwers HAA Koenen S V Kwee A. Evaluation of antenatal corticosteroid prescribing patterns among 984 women at risk for preterm delivery. J Matern Neonatal Med. (2014) 27:516–9. 10.3109/14767058.2013.821975 23826626

[24] Matlac D Jonassen S Fortmann MI Rody A Bossung V. A Question of timing: 10-year retrospective analysis on the use of antenatal steroids for imminent preterm birth. Z Geburtshilfe Neonatol. (2021) 225:493–8. 10.1055/a-1410-8379 33890265

[25] Voigt M Hesse V Rochow N Schneider KTM Hagenah HP Scholz R. New percentile values for the anthropometric dimensions of singleton neonates: Analysis of perinatal survey data of 2007-2011 from all 16 states of Germany. Z Geburtshilfe Neonatol. (2014) 218:210–7. 10.1055/s-0033-1361096 25353215

[26] Geffers C Baerwolff S Schwab F Gastmeier P. Incidence of healthcare-associated infections in high-risk neonates: results from the German surveillance system for very-low-birthweight infants. J Hosp Infect. (2008) 68:214–21. 10.1016/j.jhin.2008.01.016 18289725

[27] Shennan AT Dunn MS Ohlsson A Lennox K Hoskins EM. Abnormal pulmonary outcomes in premature infants: Prediction from oxygen requirement in the neonatal period. Pediatrics. (1988) 82:527–32. 10.1542/peds.82.4.527 3174313

[28] Bell MJ Ternberg JL Feigin RD Keating JP Marshall R Barton L. Neonatal necrotizing enterocolitis. Therapeutic decisions based upon clinical staging. Ann Surg. (1978) 187:1. 10.1097/00000658-197801000-00001 413500 PMC1396409

[29] Papile LA Burstein J Burstein R Koffler H. Incidence and evolution of subependymal and intraventricular hemorrhage: a study of infants with birth weights less than 1,500 gm. J Pediatr. (1978) 92:529–34. 10.1016/S0022-3476(78)80282-0 305471

[30] de Vries LS Eken P Dubowitz LMS. The spectrum of leukomalacia using cranial ultrasound. Behav Brain Res. (1992) 49:1–6. 10.1016/S0166-4328(05)80189-5 1388792

[31] Grobman WA Lai Y Iams JD Reddy UM Mercer BM Saade G. Prediction of spontaneous preterm birth among nulliparous women with a short cervix. J Ultrasound Med. (2016) 35:1293–7. 10.7863/ultra.15.08035 27151903 PMC5086428

[32] Gomez R Romero R Medina L Nien JK Chaiworapongsa T Carstens M. Cervicovaginal fibronectin improves the prediction of preterm delivery based on sonographic cervical length in patients with preterm uterine contractions and intact membranes. Am J Obstet Gynecol. (2005) 192:350–9. 10.1016/j.ajog.2004.09.034 15695971

[33] Owen J Szychowski JM Hankins G Iams JD Sheffield JS Perez-Delboy A. Does midtrimester cervical length ≥25 mm predict preterm birth in high-risk women? Am J Obstet Gynecol. (2010) 203:393.e1–e5. 10.1016/j.ajog.2010.06.025 20708169 PMC2947582

[34] Astiz M Heyde I Fortmann MI Bossung V Roll C Stein A. The circadian phase of antenatal glucocorticoid treatment affects the risk of behavioral disorders. Nat Commun. (2020) 11:3593. 10.1038/s41467-020-17429-5 32681096 PMC7367845

[35] Räikkönen K Gissler M Kajantie E. Associations between maternal antenatal corticosteroid treatment and mental and behavioral disorders in children. JAMA - J Am Med Assoc. (2020) 323:1924–33. 10.1001/jama.2020.3937 32427304 PMC7237984

[36] Jobe AH Kemp M Schmidt A Takahashi T Newnham J Milad M. Antenatal corticosteroids: a reappraisal of the drug formulation and dose. Pediatr Res. (2021) 89:318–25. 10.1038/s41390-020-01249-w 33177675 PMC7892336

[37] Chawla S Natarajan G Shankaran S Pappas A Stoll BJ Carlo WA. Association of neurodevelopmental outcomes and neonatal morbidities of extremely premature infants with differential exposure to antenatal steroids. JAMA Pediatr. (2016) 170:1164–72. 10.1001/jamapediatrics.2016.1936 27723868 PMC5294968

[38] Liebowitz M Clyman RI. Antenatal betamethasone: a prolonged time interval from administration to delivery is associated with an increased incidence of severe intraventricular hemorrhage in infants born before 28 weeks gestation. J Pediatr. (2016) 177:114–20.e1. 10.1016/j.jpeds.2016.07.002 27514239 PMC5037021

[39] Norberg H Kowalski J Maršál K Norman M. Timing of antenatal corticosteroid administration and survival in extremely preterm infants: a national population-based cohort study. BJOG An Int J Obstet Gynaecol. (2017) 124:1567–74. 10.1111/1471-0528.14545 28294496

[40] Battarbee AN Ros ST Esplin MS Biggio J Bukowski R Parry S. Optimal timing of antenatal corticosteroid administration and preterm neonatal and early childhood outcomes. Am J Obstet Gynecol MFM. (2020) 2:100077. 10.1016/j.ajogmf.2019.100077 32905377 PMC7469940

[41] Humberg A Härtel C Rausch TK Stichtenoth G Jung P Wieg C. Active perinatal care of preterm infants in the German Neonatal Network. Arch Dis Child Fetal Neonatal Ed. (2019) 105:190–5. 10.1136/archdischild-2018-316770 31248963

