# Supplementary figures and images for: A Timely Administration of Antenatal Steroids Is Highly Protective Against Intraventricular Hemorrhage: An Observational Multicenter Cohort Study of Very Low Birth Weight Infants
Source: Front Pediatr. 2022 Mar 16;10:721355. doi: 10.3389/fped.2022.721355 (PMC8965892; doi:10.3389/fped.2022.721355)

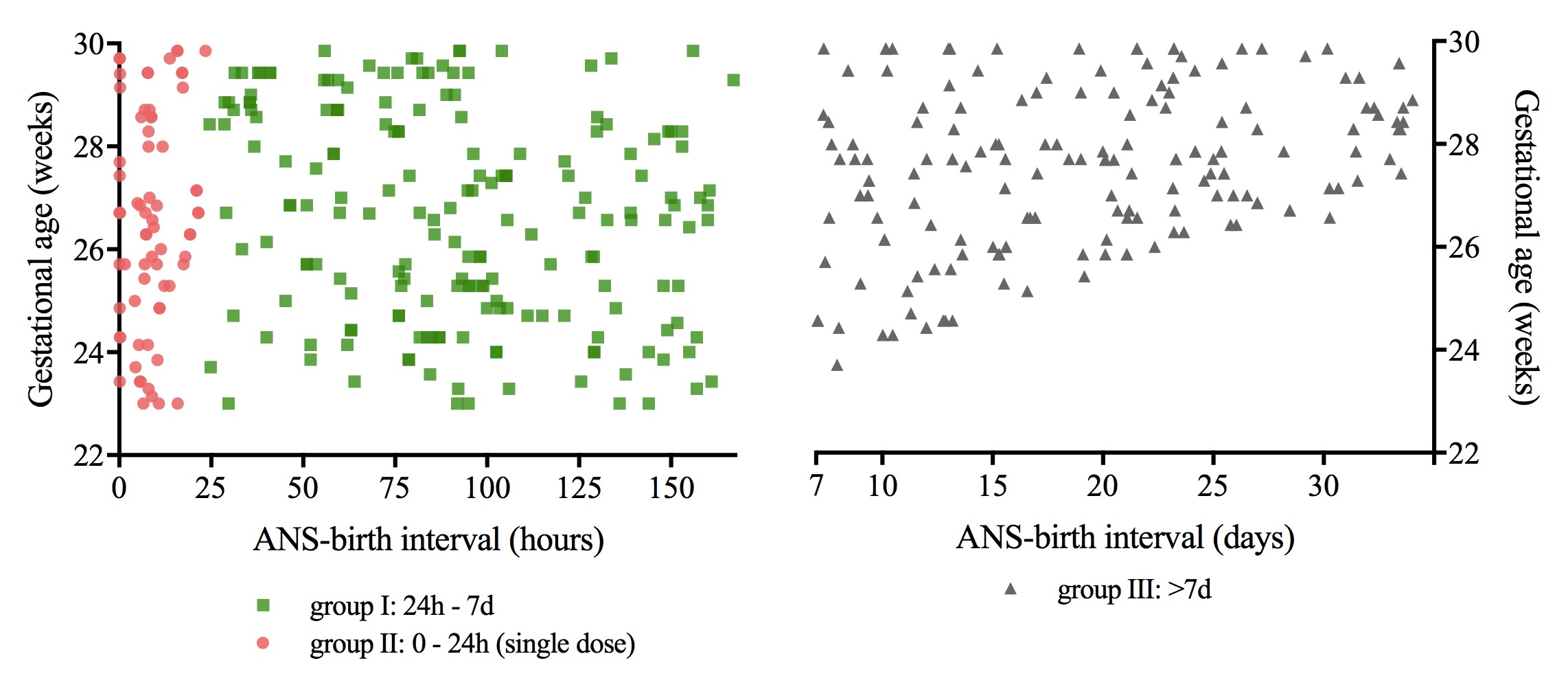

Supplement: Supplementary Figure 1 — Dot plot of ANS-birth intervals throughout different subgroups stratified by gestational age. ANS, antenatal steroids. [file Image_1.JPEG]
